# A tissue-engineered endothelial cell – smooth muscle cell arteriole-like model

**DOI:** 10.1039/d5bm00383k

**Published:** 2025-10-03

**Authors:** Ninghao Zhu, Lily Liang, Nan Zhao, Haosong Chen, Peter C. Searson

**Affiliations:** a Institute for Nanobiotechnology, Johns Hopkins University USA searson@jhu.edu; b Department of Biomedical Engineering, Johns Hopkins University USA; c Department of Materials Science and Engineering, Johns Hopkins University USA

## Abstract

Arterioles are small arteries that play a pivotal role in regulating blood flow and pressure. Co-culture of smooth muscle cells (SMCs) and endothelial cells (ECs) in a collagen fiber matrix with the correct spatial organization has been challenging. Here, we report a 3D tissue-engineered arteriole model (∼150 μm in diameter) with co-cultured confluent monolayers of SMCs and ECs. We demonstrate the structural integrity and barrier function of the model with high resolution microscopy and permeability measurements. The arteriole model recapitulates physiological contraction and dilation under pulsatile flow. In addition, perfusion with an inflammatory cytokine induces increased adhesion of immune cells on the endothelium. Together, this model provides a novel platform for studying the structure and function of human arterioles, paving the way for drug development targeting arteriole disorders.

## Abstract 2

A tissue-engineered arteriole model in a collagen I matrix with monolayers of smooth muscle cells and endothelial cells shows barrier function, pulsatile contraction/dilation, and cytokine-induced immune cell and platelet adhesion.

## Graphical abstract

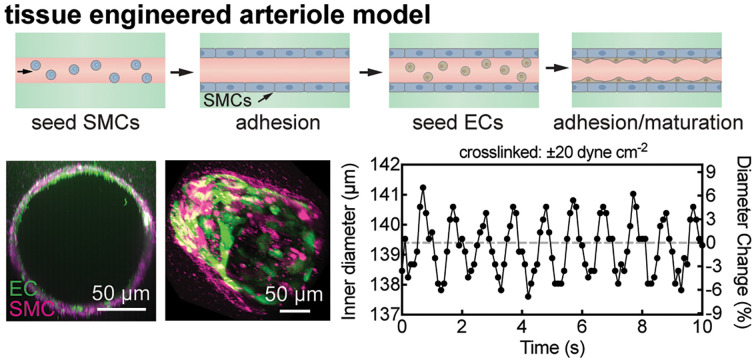

## Introduction

Arterioles are small arteries that connect arteries and capillaries. They play a pivotal role in controlling blood flow and blood pressure, contributing to about 80% of vessel resistance.^1^ Arterioles consist of tunica intima (inner layer of endothelial cells (ECs)), tunica media (middle layer of one to three layers of smooth muscle cells (SMCs)) and tunica adventitia (outer layer of collagen fibers and connective tissue). Common disorders of arterioles include arteriolosclerosis and thrombosis, leading to cardiovascular diseases, a major cause of death worldwide. A hallmark of early stage disease is inflammation in the intima and the associated accumulation of monocytes, lymphocytes, and platelets.^2^ Without intervention, plaque formation can result in stenosis and a wide range of associated pathologies. Hence, elucidating the structure and physiology of arterioles is pivotal for drug development.

The incorporation of SMCs into tubular hydrogel scaffolds has been widely studied for tissue engineered vascular grafts, however, these structures are typically >6 mm in diameter.^3–6^ In contrast, there have been relatively few reports of perfusable arteriole models.^7–9^ The major challenge has been the difficulty in reproducible seeding a confluent monolayer of ECs on a surrounding layer or layers of SMCs in an extracellular matrix at these dimensions (<200 μm). An additional challenge is optimization of medium for seeding and coculture of SMCs and ECs. Perfusable small arteries (∼1 mm in diameter) have been fabricated by bioprinting^10,11^ and sequential seeding of SMCs and ECs into PDMS tubes (no ECM).^12^ Perfusable small arteries ∼300 to 700 μm in diameter have been fabricated using a templating method with sequential seeding of human aortic SMCs and human umbilical vein vascular endothelial cells (HUVECs).^13–16^ Seeding of SMCs into the ECM prior to seeding HUVECs relies on migration and assembly of SMCs around the ECs, but has not been as successful as sequential seeding in achieving well-defined SMC layers.^13^ Self-assembly of stem cell derived SMCs and ECs in fibrin gels did not result in a well-defined SMC layer (*i.e.* tunica media), although the SMCs reportedly were important in promoting the EC network.^17^ We note that in related work, resected mouse arteries 120 μm and 220 μm in diameter have been loaded into perfusable microfluidic devices for characterization.^18,19^

Here, we report a 3D tissue-engineered arteriole microvessel with co-cultured confluent monolayers of SMCs and ECs with a lumen diameter of 150 μm. We validate the structural integrity and barrier function of the arteriole from permeability measurements. We then assess the response to pulsatile flow then show the inflammatory response to immune cells and platelets following endothelium activation.

## Materials and methods

### Cell culture and reagents

Human brain vascular smooth muscle cells from ScienCell Research Laboratories (1100, Carlsbad, CA) were cultured in Smooth Muscle Cell Medium (SMCM, 1101, ScienCell) supplemented with 2% fetal bovine serum (FBS, 0010), 1% smooth muscle cell growth supplement (1152, ScienCell), and 1% penicillin/streptomycin (P/S, 0503 ScienCell). Green fluorescent protein-expressing Human Umbilical Vein Endothelial Cells (GFP-HUVECs, Angio-Proteomie, Boston, MA) were maintained in endothelial cell medium (EC medium, Lonza Group AG, CC3202, Basel, Switzerland). Human THP-1 monocyte-like cells and HL-60 neutrophil-like cells (American Type Culture Collection, Manassas, VA) were cultured in RPMI 1640 medium supplemented with 10% FBS and 1% P/S. These cells were maintained at 37 °C in a humidified incubator with 5% CO_2_. Human platelet-rich plasma (BioIVT, Westbury, NY) was agitated at room temperature and used within 5 days of blood draw. Other reagents and materials were purchased from Thermo Fisher Scientific unless otherwise specified.

### Microvessel fabrication

SMC-EC microvessels were fabricated and maintained in a PDMS-based device as previously reported.^20^ We constructed polydimethylsiloxane (PDMS) devices featuring microchannels embedded within a collagen I hydrogel. Devices were prepared using a custom-designed aluminum mold to define channel dimensions of 1 cm length × 1.75 mm width × 1 mm height. PDMS (Sylgard 184, Dow Corning) was mixed at a 10 : 1 (base : curing agent) ratio, degassed under vacuum, poured into the mold, and cured at 70 °C for at least 2 h. Inlet, outlet, and additional access ports were created using a biopsy punch (3 mm and 5 mm diameter, 504 649 and 504 532). Each PDMS block was then irreversibly bonded to a 24 × 50 mm glass coverslip (Dow Corning) using oxygen plasma treatment (Harrick Plasma) for 1 min. The bonded chips were immediately placed on a clean surface for insertion of the template wire. A 150 μm diameter superelastic nitinol wire (8320K12, McMaster-Carr, Elmhurst, IL) was suspended through the main channel using built-in guidance channels within the PDMS. To reduce bubble formation at the PDMS–collagen interface during gel casting, the inner surface of each channel was treated with trimethoxysilane (Sigma-Aldrich, 440167-100ML) vapor for 1 h. Devices were subsequently sterilized by perfusion with 70% ethanol, then rinsed with sterile phosphate buffered saline (PBS, 10-010-049). A neutralized 7 mg mL^−1^ solution of rat tail collagen type I (Ibidi, 50205, Fitchburg, WI) was prepared on ice according to the manufacturer's protocol. The collagen was introduced into the PDMS device *via* the side ports, allowing it to fully surround the nitinol wire. Devices were incubated at 37 °C for 10 min to ensure complete collagen polymerization. To prevent delamination of the collagen gel from the PDMS walls during long-term perfusion, a 2% agarose solution (16500100) was introduced into side channels flanking the collagen matrix immediately after polymerization. After gelation, the nitinol template wire was carefully removed, forming an open, cylindrical channel (∼150 μm diameter) within the collagen matrix. The exposed lumen was then chemically stabilized by perfusing the channel with 20 mM genipin (NC9449705) at room temperature for 2 h. Genipin crosslinking improves mechanical stability and allows long-term perfusion without collapse or remodeling of the channel. Following crosslinking, residual genipin was removed by continuous perfusion with PBS at ∼0.5 mL h^−1^ for at least 12 h using a gravity-driven or syringe-pump flow system. Prior to cell seeding, the collagen lumen was coated overnight at 4 °C with 50 μg mL^−1^ human collagen IV (Sigma, C5533-5MG) and 25 μg mL^−1^ human fibronectin (Sigma, F2006-1MG) in smooth muscle cell medium (SMCM). This ECM coating mimics the basement membrane and supports both smooth muscle cell (SMC) and endothelial cell (EC) adhesion.

SMCs, labeled with CellTracker Deep Red, were then seeded into the microchannel by perfusion at a flow rate of 0.6 μL min^−1^ and a cell density of 40 × 10^6^ cells per mL in seeding medium (complete SMCM) for 30 min. The SMCs were allowed to adhere to the microchannel under static conditions for two hours at 37 °C, followed by perfusion overnight with SMC formation medium (basal SMCM supplemented with 1% P/S). The SMC microvessel was then maintained in vessel maintenance medium (VMM): SMCM supplemented with 2 ng mL^−1^ human basic fibroblast growth factor (bFGF, R&D Systems, 233FB025CF, Minneapolis, MN), 0.5 ng mL^−1^ human epidermal growth factor (EGF, AF10015100), 5 ng mL^−1^ heparin, 0.01 μg mL^−1^ recombinant human insulin-like growth factor-I (IGF-I, 10011100UG), 0.2 μg mL^−1^ bovine serum albumin (BSA, 50-121-5315), and 1% P/S. After maturation of the SMC microvessel for seven days, ECs (GFP-HUVECs) were seeded into the SMC microvessel by perfusion in EC medium at a flow rate of 0.6 μL min^−1^ at a cell density of 60 × 10^6^ cells per mL for 30 min followed by incubation for two hours under static conditions at 37 °C to promote EC adhesion on the SMC layer. The SMC-EC microvessels were then maintained in VMM. The microvessels were perfused with VMM using a gravity driven flow system with inlet and outlet reservoirs achieving a mean flow rate of 0.25 mL h^−1^.

### Image acquisition and analysis

Fluorescence images of the microvessel were obtained with an inverted epifluorescence microscope (Nikon Eclipse Ti-E, Melville, NY) using 10× objective. Fluorophores were excited by an X-Cite 120LEDBoost (Excelitas Technologies, Waltham, MA). Filter cubes (Chroma, Bellows Falls, VT) Chroma 39000, 39002 and 39007, were applied to capture the fluorescence signal from DAPI, GFP, and Cy5 channels, respectively. 3D reconstructions of the microvessel were acquired by imaging ∼400 slices with 0.4 μm in thickness on a confocal microscope (Nikon Eclipse Ti-E with CSU-X1 confocal scanner unit, Yokogawa Electric, Tokyo, Japan) using a 40× oil objective. Transmission electron microscopy (TEM) was performed to visualize microvessel ultrastructure. Microvessels were perfused with Karnovsky Fixative (15731-10, EMS, Hatfield, PA) overnight at 4 °C. Following that, microvessels were fixed in 2% osmium, 1.6% KFnCN_6_, 59 mM nacacodylate, 50 mM K_3_PO_4_, and 3 mM MgCl_2_ for 2 hours on ice, dehydrated using gradient ethanol, and infiltrated with EMbed 812 resin (14120, EMS). The embedded microvessels were sectioned into 70 nm slices with an ultramicrotome (Leica, Wetzlar, Germany), stained with 2% uranyl acetate, and imaged with TEM (Hitachi H7600, Tokyo, Japan). Images were analyzed using Fiji ImageJ.

### Barrier function

The barrier function of the microvessels was determined from permeability measurements, as reported previously.^20–23^ Briefly, the microvessels were perfused with 1 μM 70 kDa dextran labeled with CF633 (Biotium, 80141, Fremont, CA) in VMM. Phase contrast and fluorescence images were taken every 1 min for 15 min with 70 kDa dextran added at *t* = 2 min. The permeability was determined from *P* = (*r*/2)(1/Δ*I*)(d*I*/d*t*), where r is the radius of the microvessel, Δ*I* is the increase in fluorescence intensity due to the luminal filling of the perfusate, and d*I*/d*t* is the rate of increase of fluorescence is the surrounding hydrogel matrix.

### Pulsatile flow

To monitor the contraction and dilation of the arterioles under pulsatile flow, a programmable flow system (Elveflow Microfluidics, Paris, France) was used to generate a sinusoidal profile with a mean wall shear stress (WSS) of 20 dyne per cm^2^ (flow rate ≈50 μL min^−1^) and a peak-to-peak amplitude of 5 to 20 dyne per cm^2^ at a frequency of 1 Hz. The flow rate was measured at the inlet port of the microvessel using a flow sensor (MFS, Elveflow Microfluidics). The contraction and dilation of the microvessel was recorded by an inverted microscope (Nikon Eclipse TE2000) using a 10× objective with a frame rate of 10 Hz.

### Immunocytochemistry

Immunocytochemistry was performed to stain for SMC and EC biomarkers, and to quantify expression of Intercellular Adhesion Molecule 1 (ICAM-1). Microvessels were washed by perfusing with PBS and azide (Santa Cruz Biotechnology, sc-296028, Dallas, TX) for one hour and fixed by perfusion overnight at 4 °C with 4% paraformaldehyde. Microvessels were then permeabilized with 0.2% Triton-X 100 (Sigma, X100-5ML) for 30 min at room temperature and blocked with 5% BSA overnight at 4 °C. Primary antibodies for α-SMA (ab220179, Abcam), VE-Cadherin (AF938, R&D Systems) and ICAM-1 (ab2213, Abcam) were diluted by 1 : 100 in 5% BSA and perfused through microvessels for 6 hours. The microvessels were then washed overnight with 5% BSA at 4 °C, and then perfused with secondary antibody diluted by 1 : 200 in 5% BSA for 45 min in the dark at room temperature, followed by overnight washing with 5% BSA at 4 °C. Microvessels were then mounted and cell nuclei were stained with Fluoromount-G™ Mounting Medium with DAPI for at least 30 min prior to imaging.

### Immune cell adhesion assay

Endothelium activation was induced by incomplete EC seeding or perfusion with 1 ng mL^−1^ Tumor Necrosis Factor alpha (TNF-α) overnight. To assess endothelium activation, THP-1 monocyte-like cells (stained with Calcein Blue AM, C1429) and HL-60 neutrophil-like cells (stained with CellTracker Deep Red, C34565) were perfused through microvessels at a cell density of 1 × 10^6^ cells per mL for 10 min. Fresh medium was then perfused for 30 min to wash out non-adherent immune cells. Similarly, human platelet-rich plasma was stained with CellTracker Deep Red and perfused through microvessels for 10 min. Fresh medium was then perfused for 30 min to wash out non-adherent platelets. After cell adhesion, microvessels were imaged and the number of adhered immune cells was counted manually in each device.

### Statistics

All data were analyzed with Prism 10.0 (GraphPad, La Jolla, CA) and presented as mean ± SD. All experiments were conducted at least three times. Student's *t* tests were applied to compare two experimental groups. One-way ANOVA followed by Tukey's *post hoc* test was applied to compare multiple experimental groups. The statistical significance is indicated by NS (*p* > 0.05), *(*p* ≤ 0.05), **(*p* ≤ 0.01), or ***(*p* ≤ 0.001).

## Results

### Fabrication of SMC-EC microvessels

**Fig. 1:**
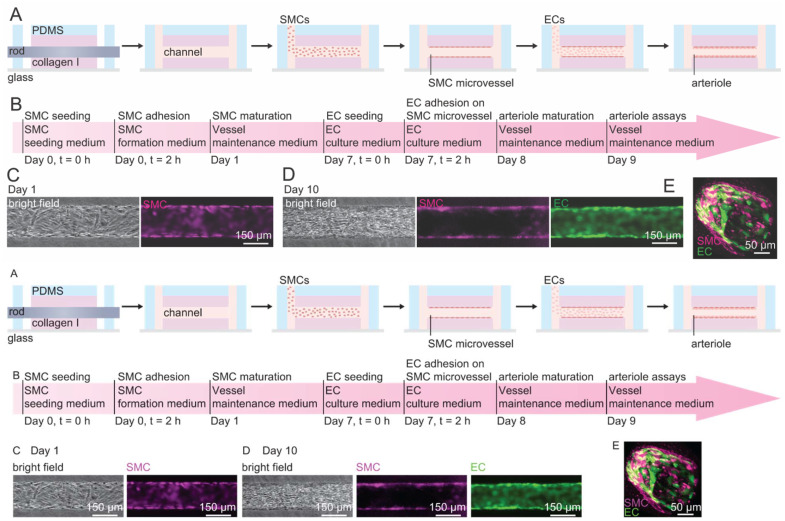
The construction of a 3D tissue-engineered arteriole model. (A) Schematic illustration of the fabrication protocol. 150 μm microchannels patterned in collagen I were sequentially seeded with SMCs and ECs to form a co-cultured confluent monolayer of SMCs and ECs. (B) The timeline of the arteriole model fabrication protocol. (C) Representative phase contrast and fluorescence images of an SMC microvessel at the mid-plane on day 1. (D) Representative phase contrast and fluorescent images of SMC-EC microvessel at the mid-plane on day 10. (E) Representative confocal images of the 3D reconstruction of the arteriole model on day 10.

Tissue-engineered SMC-EC microvessels were constructed by sequential seeding of SMCs and ECs into a 150 μm diameter channel in a collagen type I matrix to recapitulate the structure of human arterioles. The microchannel was first coated with the basement membrane proteins fibronectin and collagen IV to promote SMC adhesion. SMCs were then seeded into the microchannel and allowed to form a confluent monolayer under static conditions for 2 hours. SMC formation medium (serum-free SMCM basal medium) was then perfused overnight to remove additional cells on the SMC monolayer and inhibit overproliferation that can lead to occlusion of the microvessel. A confluent monolayer of SMCs was then achieved on day 1 after seeding, confirmed by phase contrast and fluorescence images at the mid-plane of the microvessel (Fig. 1C). SMC microvessels were then perfused with VMM for 7 days to generate sufficient basement membrane proteins for adhesion and spreading of ECs. VMM is a serum-free SMC medium supplemented with multiple growth factors and promotes SMC adhesion and monolayer formation without overproliferation. SMC medium containing FBS or smooth muscle cell growth supplement (ScienCell) all resulted in overproliferation of SMCs and eventual occlusion of the microvessel.

On day 7, ECs suspended in EC medium were seeded on the SMC monolayer in the microvessel for 2 hours. VMM was then perfused through the microvessel to maintain the SMC-EC bilayer microvessel. After 2 days (day 9), a confluent EC monolayer was formed and the SMC-EC microvessel was considered as arteriole model. The outer diameter of the arterioles was ∼150 μm. The confluent monolayers of ECs and SMCs were confirmed by phase contrast and fluorescence images at the mid-plane of the microvessel (Fig. 1D). Microscopy characterization and functional assays were performed on the arteriole model after day 9. We cultured the arteriole model for a maximum duration of 7 days (day 14).

Forming arteriole-like structures is challenging since seeding SMCs into channels requires perfusion for nutrient supply but often promotes proliferation, depending on the SMC phenotype. In addition, adhesion and maturation of SMCs typically takes longer than for ECs. The alternative strategy is to seed SMCs into the surrounding matrix, however, it is extremely difficult to promote migration to the ECs and form a well-defined layer similar to the tunica media.

### Microscopy characterization of the arteriole model

**Fig. 2:**
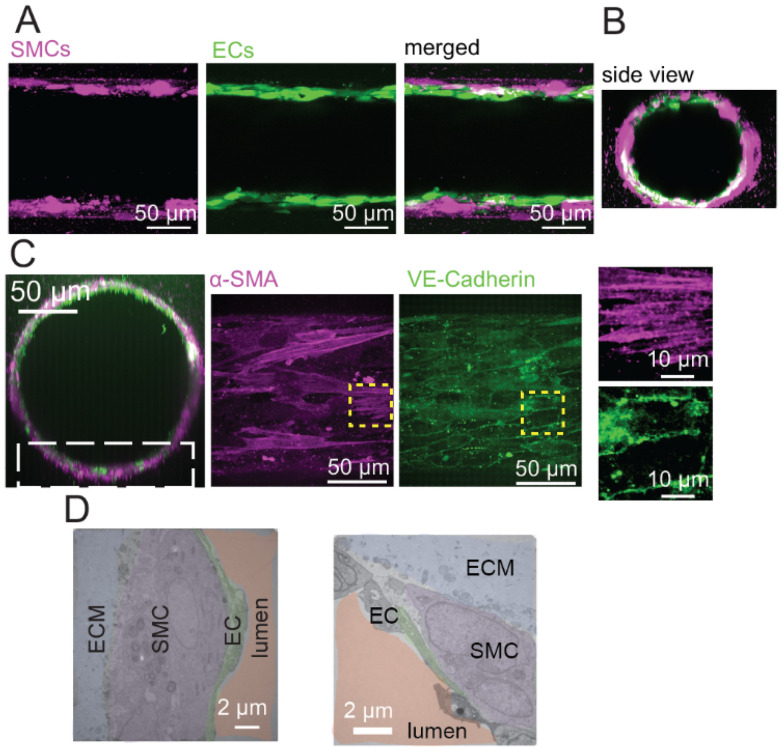
Microscopy characterization of the arteriole model. (A and B) Representative confocal images of live SMC-EC microvessel of the mid-plane (A) and side view (B) show confluent monolayer of ECs and SMCs in direct contact on day 10. (C) Immunofluorescence images of the SMC marker α-SMA and the EC junctional marker VE-Cadherin. The white box indicates the bottom plane of the microvessel corresponding to the α-SMA and VE-Cadherin images. The insets show enlarged α-SMA and VE-Cadherin images, showing actin filaments from α-SMA and cell boundary of VE-Cadherin. (D) Transmission electron microscopy images of a section of the arteriole model shows the cellular structure of the arteriole wall.

The 3D structure of the arteriole model was visualized using confocal microscopy. The mid-plane of the arteriole model (Fig. 2A) showed confluent monolayers of SMCs and ECs, with the EC monolayer luminal to the SMC monolayer. Cross-sections of the microvessels (Fig. 2B) reconstructed from the *z*-stack (Fig. 2A) further confirmed the confluent SMC and EC bilayer structure. Immunofluorescence images for the SMC marker α-SMA and the EC junctional marker VE-Cadherin confirmed the cell organization in the microvessel (Fig. 2C). The α-SMA staining showed stress fibers in the SMCs, and VE-Cadherin staining showed robust fluorescence at the EC cell boundary, indicating that α-SMA and VE-Cadherin were uniquely expressed in the SMC-EC model. We further examined the cellular structure of the arteriole wall using TEM (Fig. 2D). From cross-sectional images, the thicknesses of the SMC and EC layers were ∼8 μm and 1.3 μm, respectively, reflecting the relative sizes of SMC and EC *in vivo*, with reported thicknesses of SMC and EC layers of ∼5 μm and 0.5 μm.^1^ These results collectively confirmed the structural integrity of the SMC-EC model.

### Barrier function of the arteriole model

**Fig. 3:**
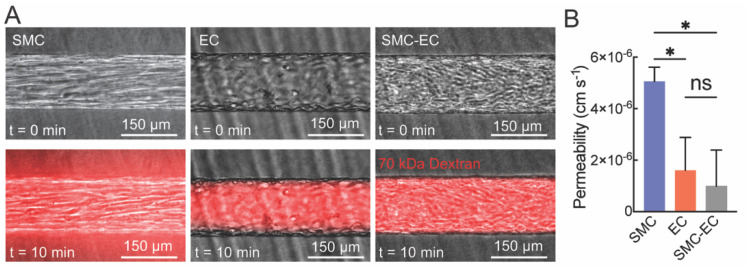
The barrier function of SMC, EC and SMC-EC microvessels following perfusion with 70 kDa dextran. (A) Real-time imaging of SMC, EC, and SMC-EC microvessels with fluorescently-labeled 70 kDa dextran. (B) Quantification of permeability across SMC, EC and SMC-EC microvessels. Data are presented as mean ± SD. One-way ANOVA followed by Tukey's *post hoc* test was used to compare the 70 kDa dextran permeability across microvessels (*n* = 3, ns not significant, *p < 0.05).

A hallmark of unfenestrated microvessels is barrier function. To assess barrier function, we determined the permeability of fluorescently-labeled 70 kDa dextran in SMC, EC, and SMC-EC microvessels (Fig. 3). The permeability of 70 kDa dextran in EC microvessels was 1.62 × 10^−6^ ± 1.33 × 10^−6^ cm s^−1^, consistent with previously reported values for HUVEC microvessels.^24^ The permeability of SMC microvessels was 5.11 × 10^−6^ ± 5.5 × 10^−7^ cm s^−1^, which is significantly higher than the EC microvessel and reflects the poor barrier function of the SMC monolayer. Interestingly, the permeability of SMC-EC microvessels was 0.99 × 10^−6^ ± 1.41 × 10^−6^ cm s^−1^, lower than for EC microvessels but not statistically significant. These results confirm the formation of a confluent monolayer of ECs on the SMCs with barrier function equivalent to ECs seeded directly on the collagen I matrix.

### Pulsatile flow in the arteriole model

**Fig. 4:**
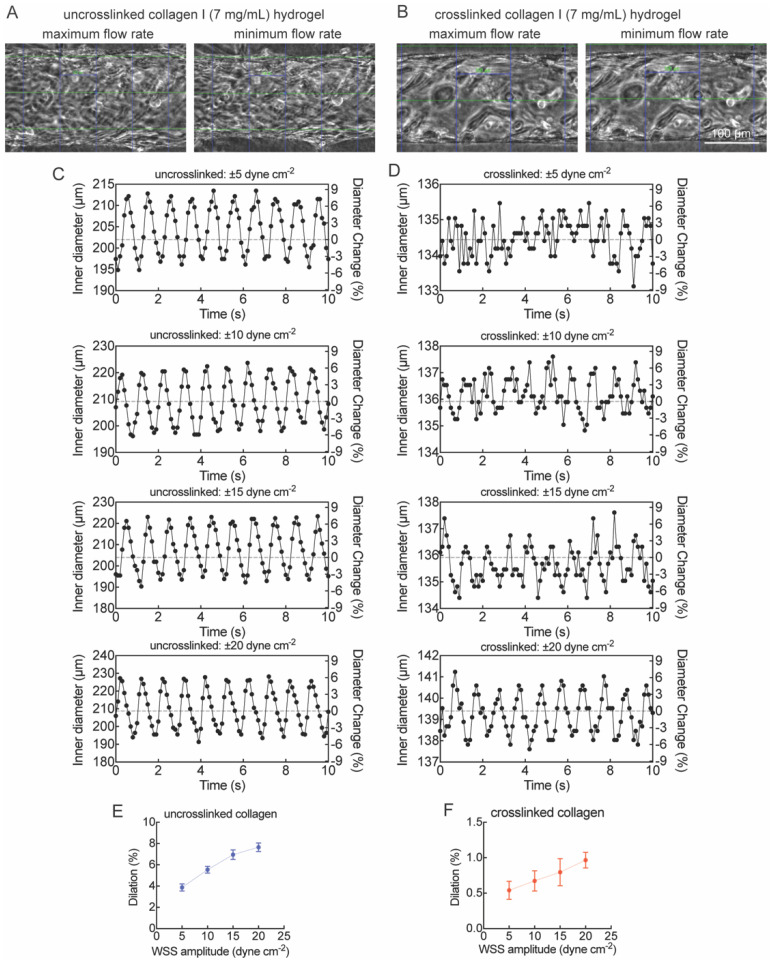
The arteriole model shows contraction and dilation under pulsatile flow. The sinusoidal profile was set to a mean WSS of 20 dyne cm^–2^ with amplitude of 5–20 dyne cm^–2^ at a frequency of 1 Hz. (A and B) Representative phase contract images of the mid-plane of the arteriole model at maximum and minimum flow rates at a peak-to-peak WSS of 20 dyne cm^–2^. (A) Uncrosslinked collagen I hydrogel (Movie 4). (B) Crosslinked collagen I hydrogel (Movie 8). (C–F) Representative plots of arteriole inner diameter over 10 s obtained from images of the periodic contraction and dilation under pulsatile flow at 10 frames per second in uncrosslinked (C) and crosslinked collagen I hydrogels (E). Profiles are shown for peak-to-peak amplitude of 5, 10, 15, and 20 dyne cm^–2^ at a frequency of 1 Hz. The dilation (D and F) was calculated by averaging the maximum and minimum diameter change in each cycle. Data are presented as mean ± SD (*n* = 10).

To model pulsatile flow, we applied a sinusoidal flow profile to visualize contraction and dilation in SMC-EC microvessels with crosslinked and uncrosslinked gels. The mean wall shear stress (WSS) in arteries is 10–20 dyne cm^–2^.^25,26^ Here we set the flow profile to a mean WSS of 20 dyne cm^–2^ with a peak-to-peak amplitude of 5 to 20 dyne cm^–2^ at a frequency of 1 Hz. The vessels showed periodic contraction and dilation corresponding to the input sinusoidal flow (Fig. 4A, B and Movies 1–8). We quantified the inner diameter of the microvessel and calculated the diameter change with respect to the mean diameter averaged over 10 seconds (Fig. 4C–F). The dilation of the arteriole model increased linearly with the amplitude in both crosslinked and uncrosslinked gels. With higher ECM stiffness (crosslinked gels), the dilation was relatively small (Δ*d* = 0.5% to 1%) compared to uncrosslinked gels (Δ*d* = 4% to 8%). These values are consistent with the typical dilation of human arteriole (Δ*d* = 3.9%) within a cardiac cycle.^27^ These observations demonstrate that our arteriole model recapitulates the physiological arteriole contraction and dilation under the pulsatile flow.

### Immune cell adhesion

**Fig. 5:**
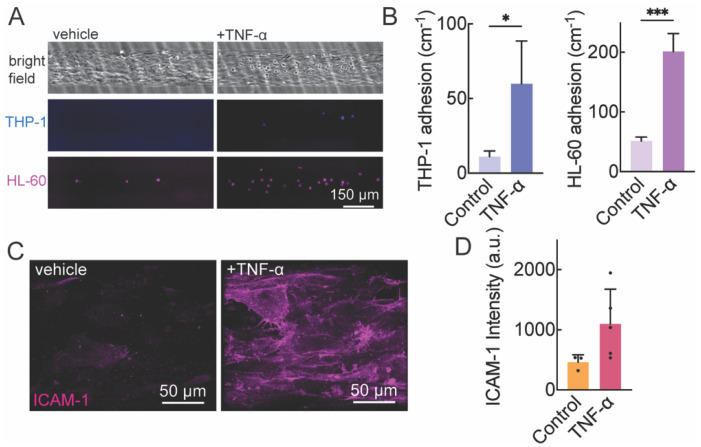
Perfusion with TNF-α induces endothelium activation in the arteriole model. (A) Representative phase contrast and fluorescence images of arterioles perfused with monocyte-like cells (1 × 10^6^ mL^−1^ THP-1) or neutrophil-like cells (1 × 10^6^ mL^−1^ HL-60) for 10 min at 12 h following treatment of TNF-α or vehicle. THP-1 (blue), HL-60 (magenta). (B) The number of adherent THP-1 and HL-60 cells was significantly increased with TNF-α treatment. (C and D) Immunofluorescence images of ICAM1 at the bottom polar plane of an arteriole model with and without TNF-α treatment. Data are presented as mean ± SD. A Student's *t* test was used to compare the difference between adherent immune cells with and without TNF-α treatment (*n* = 3, *p < 0.05, ***p < 0.001).

To investigate the inflammatory response, SMC-EC microvessels were perfused with 1 ng mL^−1^ TNF-α in VMM overnight and EC activation was assessed by immune cell adhesion and expression of ICAM-1. Immunostaining confirmed increased expression of ICAM-1 in response to TNF-α treatment. Consistent with this result, we also observed a significant increase in adhesion of both THP-1 and HL-60 cells (Fig. 5A and B).

### Human platelet adhesion on damaged endothelium

**Fig. 6:**
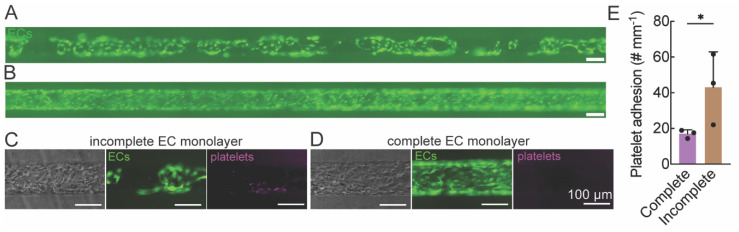
Incomplete endothelium in the arteriole model increases the adhesion of platelets on the endothelium. (A and B) Representative image of the bottom polar plane of arteriole models with incomplete (A) and complete (B) seeding of ECs (green). (C–E) The number of adherent platelets (PLT, magenta) was significantly increased in arteriole models with incomplete endothelium (C) compared to complete endothelium (D). Data are presented as mean ± SD. Student's *t* test was used to compare the difference of adherent platelets on incomplete and complete endothelium (*n* = 3, *p < 0.05).

To model the wound healing response to endothelium injury we formed SMC-EC microvessels with incomplete EC monolayers, as confirmed from fluorescence images at the polar plane (Fig. 6A). We then perfused the microvessels with human platelet-rich plasma for 10 min followed by 30 min washout to remove the non-adherent platelets (Fig. 6). We found increased platelet adhesion on the incomplete endothelium, indicating that our arteriole model is capable of recapitulating the physiological response of wound healing.

## Discussion

We have developed a human arteriole model with a single layer of SMCs around a confluent EC monolayer with a diameter of ∼150 μm. Direct co-culture of SMCs and ECs by sequential seeding in a microchannel within a collagen matrix under either constant or oscillating flow provides a robust and reproducible method for generating arteriole models. Collagen I was selected as the base hydrogel material because it is the most abundant structural protein in the interstitial ECM of connective tissues, including the tunica adventitia of arterioles.^28,29^ It provides excellent biocompatibility and is known to support both endothelial cell (EC) and smooth muscle cell (SMC) attachment and survival.^30,31^ The well-characterized fibrillar architecture facilitates microchannel fabrication and subsequent cell seeding, and it has been widely used as the hydrogel materials for *in vitro* microvessel models.^20,21,32^ To promote cell adhesion and spreading, channels were incubated with basement membrane components collagen IV and fibronectin before cell seeding. Incorporating nitric oxide assays in subsequent work will allow us to assess shear-responsive endothelial signaling, further supporting the physiological relevance of the arteriole model.^33^

Despite significant progress in vascular tissue engineering, the fabrication of perfusable, arteriole-scale models with well-defined EC and SMC bilayers remains technically challenging. Several approaches have demonstrated the feasibility of creating small artery constructs, typically ranging from 300 to 1000 μm in diameter, using methods such as bioprinting,^10,11^ sequential cell seeding into PDMS tubes,^12^ and templated collagen hydrogels.^13^ Hybrid *ex vivo* platforms with resected mouse small arteries,^18,19^ avoid the problems associated with cell seeding and provide alternative approach to model physiologically relevant processes. These collective efforts underscore the demand for 3D co-culture arteriole systems that accurately replicate native vessel architecture and function while supporting long-term perfusion and dynamic biological assays. However, even when such structural fidelity is achieved, a major biological challenge remains: maintaining stable SMC monolayers that do not undergo unwanted proliferation or phenotypic switching during *in vitro* culture.

Although advances in tissue-engineered vessels by 3D printing and templating have enabled fabrication of EC microvessel models at multiple dimensions,^7,13^ it is challenging to achieve a confluent monolayer of SMCs with sufficient basement membrane protein secretion to promote EC adhesion and spreading. In healthy human vasculature, SMCs surround the endothelium and are not in direct contact with blood. However, in response to vascular insult, SMCs can be exposed to blood serum, resulting in phenotype transformation and overproliferation resulting in neointimal hyperplasia.^34^ As a result, forming stable SMC monolayers *in vitro* is extremely challenging. Previous studies have shown that culture of SMCs with medium containing FBS promotes SMC overproliferation.^35^ Here we show that perfusion of SMC microchannels with SMC medium containing bovine serum resulted in SMC overproliferation within 12 hours of initial SMC seeding, leading to microchannels occlusion within 48 hours. Removal of serum using basal SMCM eliminated SMC overproliferation. We found that it was possible to maintain SMC channels for up to 7 days without overproliferation in basal SMCM medium supplemented with serum-free growth factors, adapted from the Smooth Muscle Growth Supplement (S00725, ThermoFisher) and which we define as vessel maintenance medium (VMM). By perfusing VMM, SMC microchannels maintained monolayers of SMCs without overproliferation for 7 days, allowing sufficient secretion of basement membrane proteins to support subsequent EC adhesion. The expression of α-SMA (Fig. 2C), indicates that the contractile phenotype of the SMCs was not altered by the depletion of serum. Formulating this novel maintenance medium, VMM was key to developing the arteriole model.

Transport measurements showed that the paracellular permeability of the SMC-EC model was the same as for the EC model, confirming that the EC monolayer formed on the SMCs maintained barrier function. Our EC-only permeability value (1.62 × 10^−6^ cm s^−1^) is consistent with previously reported values for 3D HUVEC microvessels using 70 kDa dextran,^24^ validating the technical consistency of our platform.

The model was sufficiently robust to support pulsatile flow with a mean WSS of 20 dyne cm^–2^ and peak-to-peak oscillation of 5–20 dyne cm^–2^ at a frequency of 1 Hz. We measured the inner diameter of each microvessel, calculated the corresponding flow rate and applied this flow rate to the microvessel. The dilation of the arteriole model increased with the WSS amplitude. Experiments were performed in arteriole models with crosslinked and uncrosslinked collagen hydrogels to investigate the influence of ECM stiffness on dilation. The Young's moduli of the crosslinked and uncrosslinked collagen hydrogels were 3.3 ± 0.4 kPa and 0.8 ± 0.2 kPa, respectively, as we have previously reported.^36^ The dilation of the arteriole model was lower in the stiffer ECM, indicating that the magnitude of arteriole dilation reflects the ECM stiffness (classic stretch/recoil response). These results demonstrate that our arteriole model has the potential to be adapted to model human arterioles in various organs by manipulating the ECM stiffness, and the controllable flow can recapitulate the different flow profiles in the human body. The stiffness can be modulated by tuning the gel concentration or degree of crosslinking, offering a versatile platform to mimic diverse vascular environments. For example, lower-stiffness hydrogels (∼800 Pa) may replicate adipose tissue and simulate breast cancer migration,^37^ while higher stiffness (>1 kPa) better simulates fibrotic or hypertensive vascular beds such as in atherosclerosis or pulmonary arterial hypertension.^38^ Such tunability enables disease-specific adaptation of the model to investigate endothelial dysfunction, remodeling, or SMC activation in various tissue contexts. Adjusting the level of stiffness to different degrees of crosslinking is necessary in the future work to expand the tunability of our model.

We further demonstrate that the arteriole model can simulate vascular inflammatory responses. We induced endothelium activation by perfusing the arteriole model with TNF-α. The immune cell adhesion assay showed a significant increase of the number of adherent immune cells in the arteriole model, consistent with our previous results in EC microvessels.^21^ This result indicates that our arteriole model conserves endothelium function in response to inflammation. Meanwhile, we stimulated endothelium injury by incomplete seeding of ECs on SMC microchannels. After perfusing with human platelet-rich plasma, we observed that a significantly higher number of platelets were adhered on the endothelium-injured arteriole model compared to the healthy arteriole model. This experiment models the early onset of vascular injury where endothelium loss exposes SMCs to blood serum. These SMCs are activated, expressing adhesion molecules such as ICAM-1 and producing cytokines and growth factors including TNF-α. These cytokines and growth factors will in turn upregulate adhesion molecule expression on the endothelium, resulting in increased platelet adhesion.^39,40^ We demonstrate the inflammatory response in the arteriole model with immune cells and human platelet adhesion assay, presenting a novel platform for drug discovery targeting arteriole inflammation. While leukocyte transmigration across the endothelium is a subsequent step in inflammation, we did not observe significant transmigration events within the imaging timeframe or conditions used. This can be addressed in future work using longer time-lapse imaging and higher-resolution *z*-stack acquisition to capture and quantify transmigration behavior.

While our model successfully recapitulates key structural and functional features of a human arteriole, several limitations remain. First, the smooth muscle cells (SMCs) within the collagen matrix do not exhibit circumferential alignment, a feature critical for coordinated contractility *in vivo*. We note that previous studies have accomplished circumferential alignment by seeding in PDMS channels (no ECM) with mechanical alignment cues.^12^ Second, although the expression of α-SMA suggests preservation of a contractile phenotype, we did not perform functional assays such as calcium imaging or pharmacologic stimulation to directly confirm SMC contractility. Finally, our platform is currently limited to unidirectional luminal flow and does not incorporate independent control of transmural pressure across the vessel wall, which is important for modeling interstitial transport, edema, or pressure-induced barrier dysfunction. Future designs could incorporate adjacent parallel flow channels.^19^ Addressing these limitations in future iterations will further enhance the physiological relevance of the model.

## Conclusions

In this study, we developed a co-cultured bilayer microvessel model with SMCs and ECs, constructing a perfusable, arteriole-scale lumen embedded within a collagen matrix. This tissue-engineered arteriole mimics key structural and functional features of native human arterioles, including a layered architecture, intact endothelial barrier, and tunable mechanical properties *via* ECM crosslinking. Notably, we introduced a novel, serum-free vessel maintenance medium that supports stable SMC monolayers without overproliferation, enabling basement membrane protein deposition to promote EC adhesion and function.

Our results demonstrate that ECM stiffness modulation using genipin crosslinking influences vessel compliance, suggesting the potential to model flow responsiveness in physiologic and pathologic conditions. Although direct mechanical measurements are needed in future work, this approach highlights the feasibility of tailoring biomechanical properties to simulate disease-specific vascular beds, such as those affected by hypertension, fibrosis, or neurovascular disorders.

Importantly, we also demonstrated the model's ability to reproduce endothelial inflammatory responses. Upon TNF-α stimulation, the co-cultured vessels exhibited hallmark features of inflammation, including upregulation of ICAM-1 expression and recruitment of THP-1 monocytes. These findings establish the model's utility in studying vascular inflammation under controlled 3D flow conditions.

Taken together, we demonstrated a co-cultured bilayer SMC-EC microvessel with structural and functional similarities to model human arteriole *in vitro*. This arteriole model provides a promising platform for investigating the mechanisms of inflammatory response, paving the way for drug development targeting arteriole disorders.

## Conflicts of interest

There are no conflicts of interest to declare.

## Supplementary Material

BM-013-D5BM00383K-s001

BM-013-D5BM00383K-s002

BM-013-D5BM00383K-s003

BM-013-D5BM00383K-s004

BM-013-D5BM00383K-s005

BM-013-D5BM00383K-s006

BM-013-D5BM00383K-s007

BM-013-D5BM00383K-s008

BM-013-D5BM00383K-s009

## Data Availability

The data supporting this article have been included as part of the supplementary information (SI). Supplementary information is available. See DOI: https://doi.org/10.1039/d5bm00383k.
